# Current State of Knowledge Regarding the Treatment of Cranial Bone Defects: An Overview

**DOI:** 10.3390/ma18092021

**Published:** 2025-04-29

**Authors:** Jagoda Kurowiak, Krystian Piesik, Tomasz Klekiel

**Affiliations:** 1 Department of Biomedical Engineering, Institute of Material and Biomedical Engineering, Faculty of Engineering and Technology, University of Zielona Gora, Licealna 9 Street, 65-417 Zielona Gora, Poland; t.klekiel@iimb.uz.zgora.pl; 2 Collegium Medicum, University of Zielona Gora, Licealna 9 Street, 65-417 Zielona Góra, Poland; krystianpiesik@onet.pl

**Keywords:** biodegradable materials, implants, tissue engineering, regeneration, bone tissue, personalized medicine, bone defects, DICOM

## Abstract

In this article, an analysis of the problem of treating bone defects using cranial bone disorders as an example is presented. The study was performed in the context of the development of various implant biomaterials used to fill bone defects. An analysis of the requirements for modern materials is undertaken, indicating the need for their further development. The article focuses particular attention on these biomaterial properties, which have an influence on bioresorbability and promote osteointegration and bone growth. The analysis showed the need for further development of biomaterials, the characteristics of which may be multifunctionality. Multifunctional scaffolds are those that simultaneously fill and stabilize the defect and contribute to the proper process of regeneration and reconstruction of cranial bones. Due to the complex structure of the skull and special protective functions, there is a need to develop innovative implants. Implants with complex geometries can be successfully manufactured using additive technologies.

## 1. Introduction

The human head is one of the most intricate and complex anatomical structures, integrating multiple functions such as protection of the brain, support for sensory organs, and participation in communication and mastication. In evolutionary terms, the morphology of the craniomaxillofacial skeleton has undergone significant changes over millennia, primarily influenced by factors such as biomechanical forces, metabolic demands, and brain development [1,2]. These adaptations have contributed to the current structure of the skull, which not only encases and protects neural tissue but also serves as a functional framework for vital sensory and physiological activities [3,4].

The pathologies that are localized and involve cranial structures can be caused by many factors. The most serious conditions include bone defects, complex fractures, and traumatic brain injury (TBI), which require specialist medical intervention. The importance of decompressive craniectomy (DC) should be emphasized here. DC is very often a life-saving treatment. Primary DC is used, for example, in treating intracerebral mass, while secondary DC supports the treatment of increased intracranial pressure. DC involves removing part of the skull bone to accommodate brain swelling [5,6]. Skull and brain traumas are most often caused by mechanical injuries—post-accident, congenital defects, sports injuries, violence, infections and inflammations, and tumors [7]. Attention should also be paid to cranial bone defects that result from military operations. Unfortunately, in times of war, the functioning of the medical infrastructure is limited and sometimes even impossible. Currently, there are areas around the world where there is fighting. The resulting cranio-maxillofacial defects are the result of grenades, bomb explosions, gunshots, and their shrapnel. The effectiveness of treatment in conflict countries is low. This is due to the lack of access to specialized medical equipment and personnel, and the lack of medical resources. In critical situations, one can speak of a complete lack of access to medical care [8].

Large bone defects can cause significant deformities. In cranial disorders, in extreme cases, there is a lack of protection for brain tissues. Other effects are mainly cosmetic deformities, somatic symptoms such as headaches and dizziness, and psychological symptoms, which include hypersensitivity, anxiety, and depression. The patient’s quality of life decreases significantly. Lack of treatment can cause progressive soft tissue degeneration, resulting in neurological disorders [9]. Taking into account the complex structure of the skull, the irregular shapes of the various bony structures, and the functions they perform, treatment and regeneration pose a major medical challenge [10]. Maxillofacial fractures and defects can be complex, and in addition, the intercorrelation of injuries to different skull bones may require the intervention of multiple specialists simultaneously. Hence, the cooperation of specialists in maxillofacial surgery, plastic surgery, otolaryngologists, and/or orthopedists is required [11]. The statistics relating to cranial defects differ depending on several factors. These include population variability, the particular region of the world, and diagnostic criteria. Recent reports [12,13,14,15] related to medical advances and the identification of bone defects allow a high degree of precision in treatment.

Some of the defects in the cranial bones, both after trauma and neoplastic diseases, disrupt the normal physiology of facial function. In the case of small bone defects, not exceeding 2 cm, there is the possibility of self-healing and repair. Larger defects characterized by biomechanical instability and metabolic problems require treatment [16,17]. Bone defect reconstruction and fracture treatment are very important. They allow for the healing of defects and restoration of normal facial geometry and appearance, which also affects the overall esthetics, well-being, and comfort of the patient [18].

The aim of this article is to highlight the clinical problem, requirements, and limitations associated with the treatment of bone defects located in the cranio-maxillofacial region. The paper analyzes the structure and regeneration process of bone tissue. The manuscript is a collection of information on available treatment methods, including the advantages of using implants based on polymeric and metallic meta-materials. The prepared study is intended to draw attention to technological progress in medicine, which contributes to improving the quality of treatment and offers new prospects for the future.

## 2. Anatomy of the Cranial-Maxillofacial Skeleton

**Figure 1:**
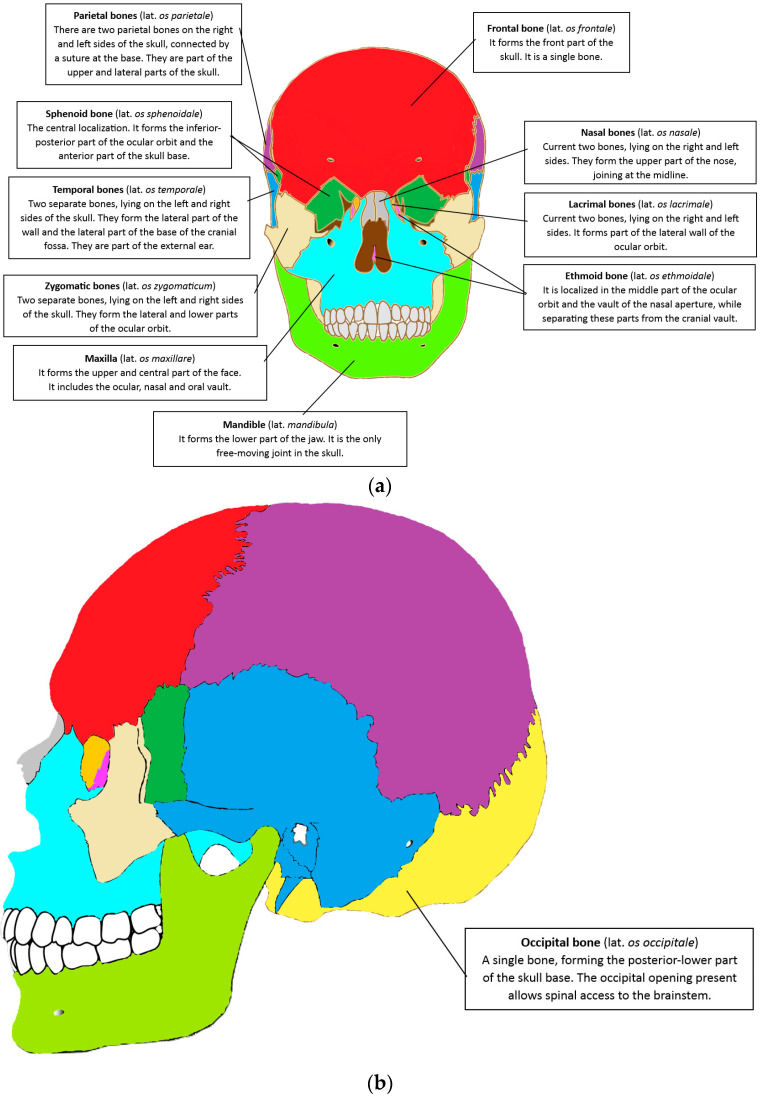
The anatomical structure of the cranial bones: (**a**) Frontal view. (**b**) Lateral view. Source: own compilation; diagram includes vector graphics (Creative Commons license).

The cranio-maxillofacial tract is anatomically made up of 22 bones, which develop in the uterus as early as during pregnancy [19]. Each of the bones that make up the skull is different in terms of its functions and anatomy. We are talking about both shape and thickness. The skull is divided into two main segments: the neurocranium and the viscerocranium [20]. The bones of the skull are connected immovably by sutures, the only exception being the mandible [21,22]. The neurocranium is made up of bones and cerebrospinal meninges, with a total of eight bones. Among them are the two parietal and two temporal bones, as well as the frontal, cuneiform, situs, and occipital bones [23]. The viscerocranium is an individual area of each person. It is what gives the characteristic appearance, which is slightly different for men and women. Depending on gender, there are some differences in bone structures [24]. For the shape of the face, 14 bones are responsible, which are a part of the eye socket and the front part of the jaw. The bones occurring singularly include the mandible and the vomer. The bones present in pairs are the bones of the palate, zygomatic, lacrimal, nasal, inferior condyles, and maxilla [16]. The structure of the cranial bones is shown in Figure 1, along with a brief description of the individual bone structures.

The bones and tissues that compose the face are special in structure and development. Their structure is slightly different from other bones found in the human body, such as the long bones and spine [25]. This is due to the embryological development of the cranial bones, which is different from that of the extra-cranial structures.

The fundamental difference and important role mentioned by Weber et al. in their work [26] is the immobilization of neuroectodermal neural cells of the cranial crest. These cells show significant differences from the mesenchymal cells of the extracranial skeletal system. Cyclic forces evoke greater responses of an anabolic nature to craniofacial sutures, as well as skull base cartilage. This means that gene expression, cell proliferation, and differentiation are mechanically regulated [26].

In the human body, bones account for up to 15% of the total body weight, and the entire bone skeleton forms the largest system in the human body [16]. The internal structure of bone is complex and consists of several materials, which include type I collagen fibers, organic compounds such as glycosaminoglycans, and inorganic compounds such as hydroxyapatite. There are two layers of bone: cortical, which exhibits greater strength, and spongy, which is slightly more flexible. The mechanics of bone is associated with their anisotropic structure and the direction of the applied force. In the work of Dewey et al. [25], one can find information on specific parameters of mechanical characteristics. The authors report that the longitudinal elastic modulus (Young’s modulus) for cortical bone is 15 to 20 GPa with low porosity of about 10%, while spongy bone has low deformability in the range of 0.1–2 GPa, but high porosity of up to 90% [25]. Other researchers’ studies indicate similar values [27,28,29].

The proper formation of the head and face is possible if the bones grow in a coordinated and integrated and, above all, in a three-dimensional manner. This allows for the proper formation and connection of bone structures [30].

The bones located near the base of the skull are quite thin, with an irregular shape. In them are located holes for nerves and vessels. Biomaterials used for implants in this area should not cause inflammatory reactions, the effects of which can negatively affect nerve structures [31]. The parietal bones of the skull located in the lateral parts (left and right sides) are thick, except for the furrow—the parietal cusp. Here, there is a significant indentation, the bone is thin. Such bone structure is of particular importance in assessing mechanical characteristics. Biomaterials used for implants for parietal bone should be porous, which increases osteointegration [32,33]. The temporal bone (left and right) is quite thin. It protects the middle and inner ear, which are responsible for hearing and balance. Temporal bone defects are unsightly and cause significant depression. Defects are most often filled with implants made of composite materials, preferably flexible, and titanium mesh. Jin et al. [34] describe the reconstruction of a temporal bone defect using titanium mesh and a silicone implant [34]. The single frontal bone is quite thick, especially within the eyebrow arches. This is the bone that gives the appearance of the face, this part is clearly visible to others. Bone defects in this area are unsightly for patients. They can be a reason for social exclusion. Implants for frontal bone defects are usually individually designed according to the patient’s needs. They are most often made of titanium mesh [35], polyetheretherketone PEEK [36], or polymethyl methacrylate PMMA [37]. In addition, Saponaro et al. [38] also pay attention to the soft tissues in the surrounding area of the facial bone defect. These tissues can directly influence the process of implant insertion and fixation [38]. In summary, the examples given of cranial bone defects require individual medical intervention. Treatment methods that are aided by rapidly developing technologies may revolutionize neurosurgery and its encompassing measures for patients in the near future.

## 3. Innovative Methods of Medical Management of Cranial Bone Injuries

**Figure 2:**
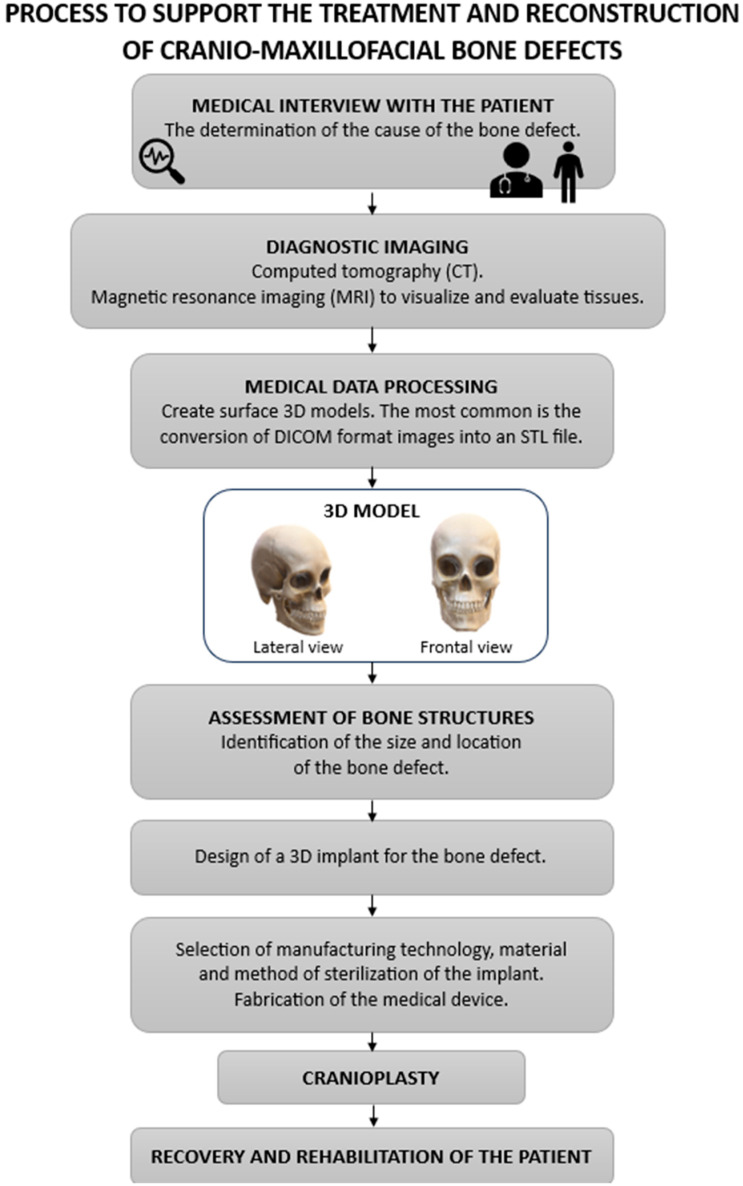
The diagram of the medical procedure in the treatment and reconstruction of cranial bone defects is based on innovative technologies. Source: own compilation; diagram includes vector graphics (Creative Commons license).

The advances in medicine are intimately related to the development of scientific fields combining the knowledge of medicine, materials engineering, biomedical engineering, and technology and digitization. The treatment of cranial bone defects uses specialized technologies: computed tomography (CT) or magnetic resonance imaging (MRI), 3D scanning, and CAD design. The development of computational modeling, which enables the creation of implants and can assist doctors both before and during surgery, should be highlighted. Before a surgical procedure, medical personnel can obtain a very accurate view of the patient’s individual bone structure. The actual reconstruction of a patient’s body structures is a revolution that can lead to the improvement of medical operations for the benefit of patients’. For doctors, it provides valuable medical information that allows them to efficiently and safely perform complex surgical and reconstructive operations. The process of assisting medical personnel in the treatment and reconstruction of craniofacial defects is presented in Figure 2.

The development of personalized implants dedicated to the issue at hand is complicated. Before starting to design an implant, it is necessary to obtain as much information as possible about the location and size of the defect, the condition of the adjacent structures, and the anatomy and function that should be preserved or restored [39].

**Figure 3:**
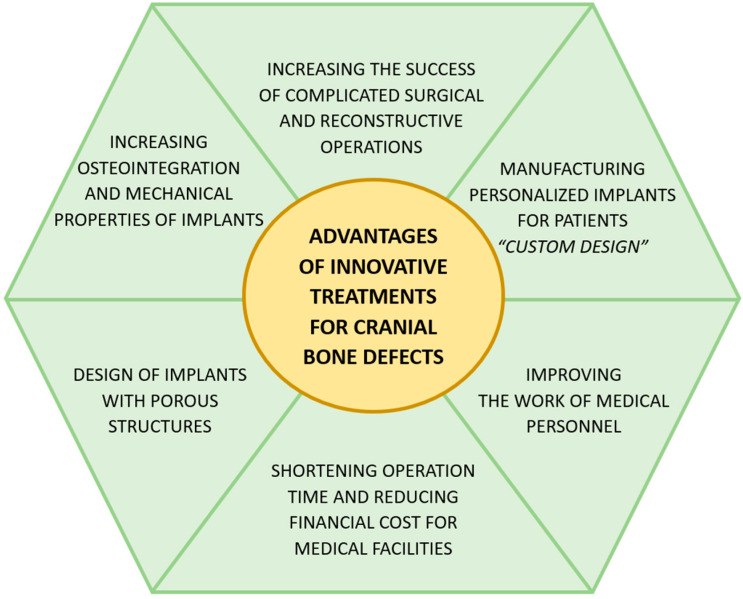
The advantages of using innovative techniques and methods for the treatment of cranial bone defects. Source: own compilation.

The advancement of technology has enabled the development of professional software and manufacturing technologies for implants and medical devices. The combination of digitization with innovative design and manufacturing methods allows the creation of personalized implants, the so-called custom design approach. This is characterized by the fact that implants are designed specifically for an individual patient. This allows for a personalized approach to a medical problem. Figure 3 shows the advantages of using innovative and modern techniques and methods to treat bone defects.

The diagnostic methods based on imaging (CT, MRI) are constantly improved. These are methods that allow obtaining both cross-sectional images (2D) and spatial images (3D) of the bone structures and tissues under examination, the so-called DICOM images (from Digital Imaging and Communications in Medicine). The medical field is taking advantage of the capabilities of these methods and using additional programs that allow the segmentation of medical images. Currently, for surgical–reconstructive procedures, computer-assisted imaging is being used along with appropriate analytical and navigation tools [40,41]. This is one of the main standards in medical institutions. The specialized software allows combining 3D images of the patient’s bone structures with the designed implants. In this way, even before the operation, the surgeon can obtain a visualization of the possible end result. In addition, professional software enables numerical analyses that simulate the behavior of implants and bone when various loads or impacts occur. Such visualizations make it possible to determine whether the bone-implant fit is correct, whether stability of bone structures has been achieved through the fixation used, and to eliminate possible complications and adverse events [40].

The 3D printing technology is one of the most popular methods of manufacturing implants. There are such technologies as follows: FDM (from Fused Deposition Modeling) and SLA (from Stereolithography). Its primary advantage is the relatively simple principle of operation. It seems that 3D printing can successfully deal with the challenges of reconstructing bone defects [41].

## 4. Methods of Treatment and Reconstruction of Cranial Bone Defects

The prominent methods of treatment and repair of cranio-maxillofacial defects are bone grafts and reconstructions using implants. They are divided into four basic types, depending on the origin of the tissues: autograft, allograft, the less commonly used xenograft, and the currently very popular natural or synthetic replacement materials—implants. These methods are regarded as standards and gold measures in the reconstruction of cranial bone defects [11,42,43].

An autologous transplant involves the use of the patient’s own tissue. In cranioplasty, this typically entails harvesting bone from the iliac crest. When the graft includes a direct blood supply via vascular anastomosis, it is referred to as a vascularized graft. In contrast, non-vascularized bone grafts—such as those harvested from the fibula—do not include such vascular connections. Vascularized bone grafts generally demonstrate higher success rates due to the presence of viable osteoblasts, which enhance osteointegration and osteogenic potential at the implantation site [3,43]. However, autologous grafts are associated with certain drawbacks, including donor-site pain, infection risk, potential bone deformity, discomfort, vascular injury, and nerve palsy [3,25]. Another limitation is the restricted availability of suitable donor bone tissue [44].

The allogeneic transplant can be from a living or deceased donor. After collection, the material is subjected to strict medical procedures that determine how it is stored and implanted in the patient. This type of transplant is more complicated than autologous transplantation, mainly because of the need to minimize the impact of genetic material and pathogens from the donor on the organism of the recipient. This can minimize the risk of disease transmission. The disadvantages of allograft are the risk of graft failure and infection, and a lower rate of osteogenicity [25,43].

Xenografts, which are transplants derived from a donor of another species, are most commonly obtained from porcine or bovine species. Their availability is significantly greater than that of autografts or allografts. However, their use requires strict medical processing to reduce the risk of disease transmission, particularly zoonotic diseases. All such transplants require rigorous medical procedures, due to increase the risk of zoonotic disease transmission and a higher probability of graft rejection [43].

The development of an effective therapeutic method is essential for the health of patients and important for economic reasons. The substitute natural and synthetic materials that form implants are a modern and progressive method of treatment of skull bone defects, including large and complex ones.

## 5. Cranioplasty and Bone Defect Reconstruction

The cranioplasty is a routine neurosurgical procedure that is performed, among other things, after a craniectomy to repair defects in the skull. As a reminder, a craniectomy is an invasive surgical procedure that involves opening up the skull by removing part of the bone without putting it back in its original place. Other causes requiring cranioplasty include the removal of cancerous tumors, traumatic defects, or congenital defects. The main task of cranioplasty is to restore the brain’s protection, improve the brain’s bioelectrical function, and support the processes occurring in the brain, such as blood flow and control of abnormalities in the cerebrospinal fluid. This ultimately has a positive effect on improving patients’ neurological and motor functions. In addition, it fulfills cosmetic and rehabilitative aspects, which contributes to speeding up and improving rehabilitation [33,45,46,47,48,49]. The important results of cranioplasty for patients are primarily an improvement in the appearance and symmetry of the skull, which translates into psychological aspects and human contacts and relationships [50,51]. Unfortunately, in some cases, it is not possible to perform cranioplasty. The main contraindications are hydrocephalus and significant cerebral edema, infections, and sometimes there are age restrictions for patients. This applies primarily to children who are under 3 years of age. In their case, there is a chance that the skull will heal on its own. Additionally, it is accepted that most cranial bone reconstructive procedures are performed approximately 6 months after injury or illness [47,51].

**Figure 4:**
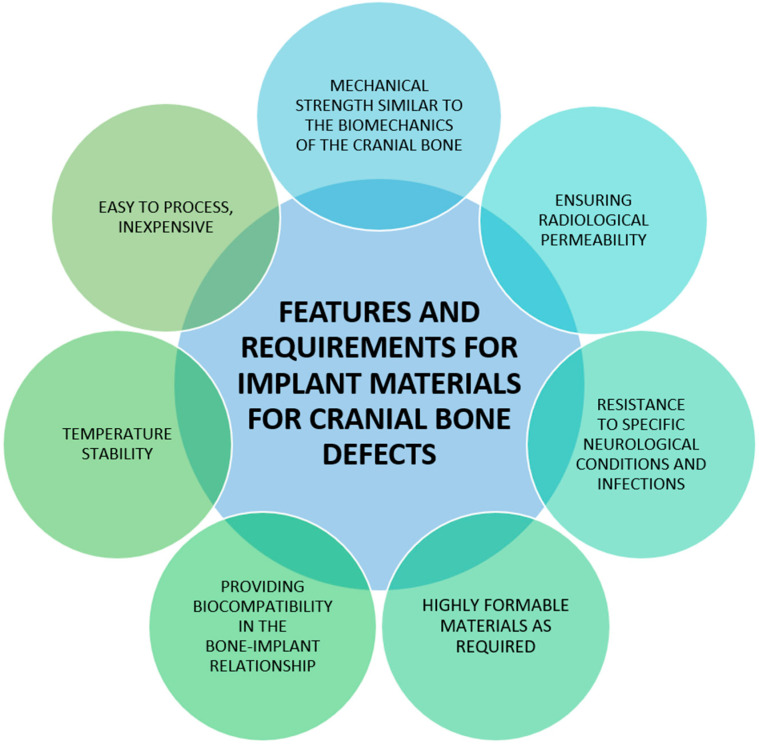
The criteria for the selection of materials dedicated to the treatment of cranial bone defects. Source: own compilation.

The reconstruction of cranial bone defects that result from trauma or disease is a significant medical challenge. According to the size and complexity of the defect, the reconstruction can be medium (up to 110 cm^2^ of the defect) or large (more than 110 cm^2^) [48]. However, regardless of the size of the defect, it is important to properly design the implant and select the right material. As a manufacturing technology, 3D printing is most often used, which is very popular in bone tissue engineering and regenerative medicine. The advantage of 3D scaffolds over other treatment solutions is primarily due to their unique properties. Three-dimensional porous structures can promote cell proliferation, which has a positive effect on tissue regeneration [52,53,54,55]. Material selection criteria, features, and requirements are shown in Figure 4.

The materials used can be durable or biodegradable. The main advantages of biomaterials are their consistent quality, high sterility, and general availability. Unfortunately, so far it has not been possible to develop a single effective solution that meets all the requirements for implants. This is due to the limitations encountered, both mechanical and structural, and those related to biological issues, i.e., the risk of infection or improper integration of the implant into tissues. This raises the need for further research.

The most effective and successful treatment can be provided when the implant is biologically similar to the tissue. We are talking about biocompatibility and the high properties of osteointegration, osteoinduction, osteogenicity, and osteoconductivity [44,56,57,58]. These properties influence the immune response of the organism to the implant and increase the degree of integration into the tissue environment [44]. During the design of scaffolds, it is necessary to take into account the correct matching of the implant geometry to the bone defect.

Implants for cranial bone defect reconstruction can be made of materials such as metals, polymers, and bioceramics [59].

The repair of bone defects is a multistep and complex process consisting of inflammation, angiogenesis, and osteogenesis. It is important to both promote bone regeneration and prevent infection. In order to accelerate the remodeling process of bone defects and reduce the occurrence of adverse phenomena that impede the course of regeneration, the concept of multifunctional scaffolds was developed. The multifunctionality of these scaffolds is that, in addition to the primary stabilizing function of the bone defect, they additionally play a role in supporting the regeneration process. Thus far, there have been numerous articles describing concepts related to the use of multifunctional implants, as well as applied in clinical practice.

The first is the possibility of applying antibiotic therapy or using bacteriostatic material to create a scaffold. The scaffolds can be enriched with an antibiotic, which, released gradually, allows for the prevention of post-therapeutic infections at the surgical site or is able to combat those that have already occurred. This type of scaffolding allows for a high local concentration of the drug, which, with the simultaneous administration of the antibiotic systemically, offers the prospect of greater treatment efficacy [60].

The next concept is to provide scaffolds with agents that stimulate bone tissue regeneration. The use of an osteoinductive substance embedded in the bioscaffold makes it possible to accelerate bone remodeling by, among other things, inducing osteogenic cells and increasing the speed of their migration process to the site of pathology [61].

There have been publications showing that it is possible to combine anti-infective properties with bone regeneration-promoting properties to obtain even better treatment effects. Such solutions open up new possibilities related to the creation of multifunctional scaffolds [62,63,64,65,66,67,68].

Research by Rather et al. [69] has highlighted the important role of angiogenesis (vasculogenesis) in the process of bone regeneration. Their dual-function scaffolding concept relies on the induction of osteogenesis and angiogenesis as a key component of the bone regeneration process. They indicate that vascularization helps re-establish blood supply to the resulting bone defect, and thus supply the necessary substances for its reconstruction [69].

Tan et al. [70] presented the concept of a scaffold that combines the promotion of osteogenesis with the simultaneous inhibition of tumor cell proliferation. This type of scaffold can be used in the treatment of cancer-induced bone loss [70].

### 5.1. Most Popular Materials Used in Cranioplasty

#### 5.1.1. Titanium (Ti)

The most common metal used for bone implants is titanium (Ti) and its alloys, most commonly Ti6Al4V [71]. However, the occurrence of this element in the human body should not exceed 0.21 mg/1kg body weight [72]. The advantages of this metal include good mechanical strength, biocompatibility in the living body environment, and potentially low risk of tissue inflammation [73]. However, the disadvantages of Ti that impact treatment efficacy are related to its high density in the range of 4.37–4.56 g/cm^3^ [74]. The possible risks are erosion of the soft tissues covering the implant, which can result in its exposure, the occurrence of allergies to the metal, and the formation of deformations of the material under the influence of heat and external forces [73,75]. A concentration of Ti that is too high in the body negatively affects endothelial homeostasis and exacerbates inflammation of surrounding tissues, which unfortunately leads to the imbalance between osteoblasts and osteoclasts [72]. Avery et al. [76] also point to the serious problem of bone collapse and stress compensation. These are issues related to differences in the mechanical strength of titanium and bone [76].

The other example of a Ti-based biomaterial is nitinol (NiTi). Nitinol is an alloy of nickel and titanium. NiTi exhibits shape memory and is a superelastic material. NiTi has good mechanical properties, is biocompatible, and shows high corrosion resistance [77,78]. Nitinol is used in medicine as a material for bone implants, among other applications. The main disadvantage of NiTi is the release of Ni ions from the implant surface into the organism. Unfortunately, nickel ions can cause inflammation and allergic reactions [77].

#### 5.1.2. Polyetheretherketone (PEEK)

Polyetheretherketone (PEEK) is a thermoplastic synthetic polymer that is successfully used to replace titanium implants due to its unparalleled properties. The PEEK has very good mechanical properties [79], which are similar to the mechanical characteristics of cortical bone [80], which can promote better regeneration [81]. In addition, it exhibits anti-corrosive properties [82] and is a radiolucent material, which allows the process of bone ingrowth into the implant to be observed by radiography [76,83]. Jindal et al. [84] emphasize that PEEK is bio-inert, as it does not release toxic products into the body, while it may slightly reduce the success of osteointegration [84]. An extensive study of the treatment of craniofacial defects with PEEK implants is described in the work of Cárdenas-Serres et al. [85]. In their study, the authors evaluate the effectiveness of PEEK implants, which are tailored to the individual craniofacial reconstruction needs of patients [85].

#### 5.1.3. Polycaprolactone (PCL)

Polycaprolactone (PCL) is a semi-crystalline polymer from the aliphatic polyester group. It is characterized by low longitudinal elastic modulus (Young’s modulus) and limited tensile strength. As suggested by Hashim et al. [86], it is possible to improve the mechanical properties of PCL by introducing various additives [86]. PCL is a biodegradable material with a degradation time of 2 to as much as 4 years, depending on the molecular weight [87]. Due to its biocompatibility and stability in tissue and body fluids [88], it has been successfully used in tissue engineering as a system for controlled drug release [89,90], as implants for bone tissue defects [91,92,93], and in reconstruction of craniofacial defects [94,95,96].

#### 5.1.4. Polymethyl Methacrylate (PMMA)

Polymethyl methacrylate (PMMA) belongs to the polymerized acrylic acid ester group [97]. Its mechanical properties are similar to those of cortical bone. PMMA is a highly ductile material that is easy to shape and adapt to specific requirements. It is a biocompatible material that is permeable to radiation, chemically inert, and relatively inexpensive [98]. The main disadvantage of PMMA is the presence of monomer residues from the polymerization process, which can negatively affect cells. The surface of implants made from PMMA is not porous, which can hinder osteoconduction [99].

#### 5.1.5. Hydroxyapatite (HA)

The advantage of hydroxyapatite (HA) over other materials is that it is a natural component occupying about 70% of bone mass. Implants based on synthetic HA show high biocompatibility in a living tissue environment, which promotes cell growth and new bone formation [100,101]. Also noteworthy is macroporous hydroxyapatite (mp-HA), which has recently been used in neurosurgery. The rough surface primarily promotes osteointegration within the cranial vault. The integration and migration of osteoblasts between the cranial vault and the mp-HA implant positively influence proliferation and the process of bone regeneration and reconstruction [102,103].

#### 5.1.6. Poly(L-co-D,L-lactide) (PLDLA)

Poly(L-co-D,L-lactide) (PLDLA) is an amorphous polymer of the lactide group. It has good mechanical properties [104]. The structure of PLDLA contains about 30% of d, l-lactic acid residues [105]. Its molecular weight is in the range of 10^5^ g/mol, and its glass transition temperature is between 55 and 60 °C. The degradation time of PLDLA depends on the molecular weight and is about 6–7 months [106].

Table 1 shows selected articles that describe research on implants for the treatment of craniomaxillofacial defects.

In Table 1, an analysis of the state of the art for the implants used to treat cranial bone defects is presented.

## 6. Innovations in Craniomaxillofacial Implants with the Addition of Hydrogel Materials

The metal implants or those based on single-component polymers may be an insufficient solution for progressive and more complex defects in the skull bone. Therefore, new and innovative solutions based on multi-component and multifunctional scaffolds are being sought.

The future in the treatment of craniomaxillofacial defects may be hydrogels, which are slowly gaining popularity in neurosurgery and maxillofacial surgery. The hydrogels can increase treatment efficacy due to their high bioactivity, which promotes stem cell proliferation and differentiation [116]. The hydrogels are characterized by a three-dimensional structure that is capable of absorbing body fluids, making them similar in structure to the extracellular matrix [117,118]. Alipour et al. [116] attempted to fabricate a PEEK-based implant with the addition of aldehyde-cellulose hydrogels, nanocrystalline and silk fiber. In the study, they evaluated the ability of the multi-component implant to regenerate craniofacial bone and periodontal areas. In vivo studies were performed on animal models of rats with significant bone loss. As the authors suggest, the introduction of hydrogels into PEEK implants had a positive effect on bone regeneration [116]. Wang et al. [119] developed a cranial implant based on PCL, nanohydroxyapatite (nHA) and a bioactive glycopeptide hydrogel. In the study, they proved that the hydrogel promoted the growth and differentiation of stem cells. The mechanical properties of prepared scaffolds, that as tensile and compressive modulus were range of 180–506.6 MPa and 44.9–56.43 MPa. After 12 weeks of testing, a bone tissue repair process was observed. The microenvironment created by the presence of the hydrogel promoted osteoinduction and biochemical processes that accelerated the bone repair process [119]. Dong et al. [120] developed a multifunctional hydrogel-based material that can significantly contribute to the effective treatment and repair of cranial bone defects. The studied hydrogel material was made from dopamine, sodium alginate, carboxymethylchitosan, calcium ions, nHA, and magnesium oxide. The authors report that the compressive mechanical properties for prepared hydrogels of 4.42 ± 0.5 MPa. Analyses showed that the developed material is biocompatible, capable of promoting cell proliferation processes, and exhibiting repair and regenerative properties [120]. Other reports describe a material made of bioactive poly(ethylene glycol) hydrogel, chondroitin sulfate, and collagen. The material produced is characterized by high porosity, which promotes cell proliferation, migration, and adhesion. The mechanical properties of hydrogels were evaluated by rheological analysis. The critical strain values for the four hydrogel groups tested were approximately 35.8%, 85.5%, 87.8%, and 21.2%. Its biocompatibility and controlled biodegradation make its use in the reconstruction and repair of significant cranial bone defects a promising therapeutic solution [121].

## 7. The Implant-Biofilm Relationship in the Perspective of Successful Treatment

Any implant placed in the body is considered a foreign element by the host organism. The new implant surface becomes an attractive site for both recipient cells and microorganisms. In order to achieve effective treatment of bone defects, the implant must be able to be tolerated by the patient’s cells. In this case, success is dependent on the action and activity of the biofilm [122].

The biofilm is a structure formed by a community of bacteria or fungi with a specific microenvironment isolated inside. It is composed of an extracellular matrix of deoxyribonucleic acid (DNA), polysaccharides, proteins, and lipids, which attach to the surface of implants in numerous clusters. The biofilm is most often surrounded by an extracellular polymeric substance (EPS), which plays a protective role [123]. It is estimated that after implantation with various biomaterials, the rate of observed infections can exceed as much as 12% [122,124]. The polymeric materials, composites, and metals and their alloys used for implants are all susceptible to negative effects from the bacterial environment. Bacterial infections diagnosed after implantation of an implant can cause serious consequences for the patient’s health. Unfortunately, very often the bacterial biofilm is resistant to antibiotic therapies [125]. The most common group of microorganisms, the pathogenic agents responsible for biofilm formation, include Gram-negative bacteria such as *Staphylococcus aureus*, *Staphylococcus epidermidis*, and *Pseudomonas aeruginosa* [125,126,127,128]. Serious diseases caused by biofilm include chronic pneumonia and respiratory infection. The biofilm bacteria entering the bloodstream can cause severe and secondary infections, such as bacteremia or septicemia [129]. Learning about the pathogenesis of the biofilm formed by microorganisms and its identification is important in medicine, as its effects can impede bone tissue regeneration and reconstruction processes. Biofilm can delay or prevent the complete healing of surgical wounds. Additionally, once formed, the bacterial structure is difficult to remove and often forces reoperation to remove the implant [123,125].

The body’s defense reactions (immune defenses) to the activity of microorganisms can further negatively affect the bone structures adjacent to the implant by destroying them [129].

**Figure 5:**
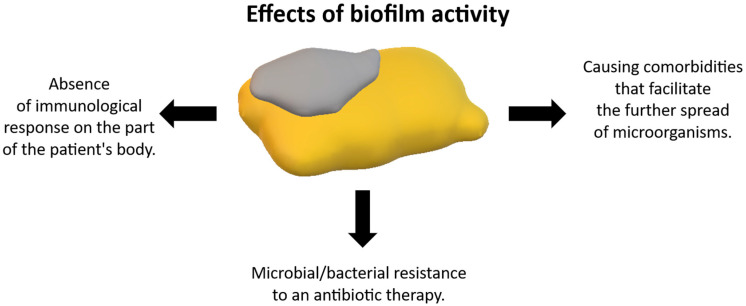
Effects of biofilm action and activity. Source: own compilation.

The first step (I) involves the attachment of free-living bacteria, so-called planktonic bacteria, to the surface of the scaffold. Two phases are characteristic of this stage, a reversible and an irreversible phase, which is more biologically stable [122]. The initial reversible phase is one for which physical forces such as gravity, diffusion, van der Waals forces, surface electrostatic charge, and bacterial cell motility are responsible. The reversible fundamental phase, on the other hand, is one in which the distance between the bacterium and the substrate is reduced (from 150 nm to ~3 nm). Hydrogen bonds, which stabilize the connection, are responsible for this action. In the irreversible phase, bacteria multiply with the simultaneous formation of glycocalyx. At this stage, the bacteria become permanently attached to the substrate, and the speed of this phenomenon depends on the bacterial strain and environmental conditions [130]. The multiplication of bacteria in step (II), that is, their accumulation and attachment to the substrate of the biomaterial is based on mutual bacteria-bacteria adhesion. The next step (III), or maturation, involves the growth of the three-dimensional structure of the biofilm. During this stage, the interior of the biofilm can be divided into numerous microenvironments that differ in their conditions (e.g., oxygen concentration or amount of nutrients), which translates into a diversity of functions of the bacteria residing there. The different conditions allow different strains of bacteria to nest and grow within a single biofilm. In addition, close contact between cells induces the process of horizontal gene transfer, which contributes to increasing the genetic diversity of the population with the possibility of developing antibiotic resistance. The final phase of biofilm formation is step (IV), characterized by the detachment and separation of bacteria from the implant surface. In this step, microorganisms are released spontaneously or due to an external stimulus, which contributes to the colonization of pathogens [122,130]. This activates the mechanism of spreading infection. The bacteria can move in the body of an infected person from one area to another. As time passes and the biofilm ages, there is a decrease in bacterial activity. As a result, this leads to the spread of microorganisms to further free areas of the implant [131]. Figure 5 shows the effects of biofilm action and activity.

### 7.1. Factors That Affect Biofilm Formation

The formation of biofilm is promoted by many factors, both by the environment and by the human body, and also applies to the surface of implants. The nature of a material’s surface plays an important role in biofilm formation. Rough surfaces show increased bacterial adhesion while increasing the size of bacterial clusters. In addition, the surface-to-water ratio translates into biofilm formation. Materials characterized by the highest hydrophobicity were most conducive to this phenomenon [132]. The appearance of bacteria can cause danger to the organism. The warning signals generated force the formation of a stable microenvironment in the form of a biofilm [133,134]. These variables include, but are not limited to, temperature, pH, amount of available food, presence of antibiotics, adhesion inhibitors (surfactants), oxygen concentration, and osmolarity [133,135,136].

### 7.2. Importance of Biofilm in the Context of Treatment

The formation of biofilm on 3D scaffolds implanted in a patient is still a high risk regardless of careful aseptic and antiseptic protocols. Elderly, chronically ill patients are particularly susceptible to all kinds of postoperative complications. The resulting biofilm is a potential source of sepsis—an imminently life-threatening condition. Therefore, it is important not only to treat but also to prevent the formation of this structure. In addition to life-threatening conditions, prolonged treatment of surgical wounds represents a major cost to public health care [137,138].

The biodegradable 3D scaffolds are a potential environment for bacterial growth. In order to avoid all the possible consequences associated with this, work is being undertaken that may provide effective methods to combat microbes.

The therapy of bone defects using biodegradable 3D scaffolds is an innovative method that carries many advantages. One of them is the reduction in the number of surgeries needed to be performed. Once implanted, the scaffold degrades after fulfilling its role, eliminating the need for further procedures to remove it. However, the application of such a scaffold also carries the risk of infection and biofilm development on the biodegradable structure. To minimize the risk, the idea of dual-function scaffolds was introduced. In addition to stabilizing the defect site, this scaffold can release various types of pharmaceuticals, including antibiotics, to limit bacterial growth. Another great advantage of this solution is the ability to achieve a high, effective local drug concentration, which is not always possible with systemic antibiotic delivery [64,68,139].

In addition to antibiotics, scaffolds can also be provided with osteoinductive substances to promote bone tissue growth and reduce the time it takes to repair a bone defect, especially in complex injuries [68].

## 8. Conclusions and Further Perspectives

Cranio-maxillofacial defect reconstruction remains a rapidly evolving field. This advancement is driven by innovative technologies as well as constantly evolving surgical solutions. Advances in tissue engineering, regenerative medicine, and biomaterials have enabled scientists and medical professionals to improve the treatment of defects in the craniofacial area. However, it is important to acknowledge the limitations and challenges that remain unresolved. Long-term clinical studies are still required to assess the behavior of implants in relation to surrounding tissues and the overall impact on the human body. An important aspect for further consideration is the enhancement of osteointegration and biocompatibility, which are important for the proper function of implants in load-bearing structures.

Research on the development of ideal biomaterials for craniofacial implants indicates the need for continued advancement and expansion of knowledge in the field of interaction of biomaterials with the tissue environment. An analysis of the achievements to date allows us to conclude that a promising direction for further development in this field is certainly research developing scaffold technology and including modern manufacturing techniques using additive 3D printing methods. Despite the already well-known materials such as bioceramics, hydroxyapatite, certain polymers, and metals, challenges related to biological response, osteointegration, and biocompatibility problems are still encountered. Treating defects in the craniofacial region is complex. Cranioplasty implants must exhibit sufficient mechanical strength and stiffness, and adapt to the specific loading conditions present in the skull. To date, an ideal 3D scaffold for the reconstruction of craniomaxillofacial defects has not been developed.

The current state of knowledge regarding the treatment of cranial bone defects has significantly advanced, incorporating a range of both traditional and innovative approaches. In our opinion, the primary focus should be on developing materials with improved properties such as enhanced biocompatibility, increased mechanical strength, greater flexibility, and the ability to promote tissue regeneration. Cranial bone defects, often resulting from trauma, congenital conditions, or surgical procedures, pose significant challenges in terms of reconstruction and restoration of both function and esthetics. The primary goal in treating these defects is to restore the structural integrity of the skull, protect the underlying brain tissue, and improve the patient’s quality of life.

Additionally, there is a growing focus on developing materials that integrate more effectively with the surrounding bone and soft tissues. Furthermore, there is a need to create materials capable of adapting to the natural healing processes of the body. Another key aspect is the development of materials that are lightweight and durable, aiming to minimize the risk of complications and improve patient outcomes. Moreover, these materials should aim to reduce the risk of infection, lower the likelihood of implant rejection, and allow for more precise and customized treatments.

Customized treatments are crucial in the context of cranial bone defect repair for several reasons. First, each patient’s anatomy is unique, and a one-size-fits-all approach may not provide the best functional or esthetic outcomes. Customized treatments using 3D-printed implants allow for precise matching to the individual’s cranial structure. This level of precision enhances surgical accuracy, reduces the risk of complications, and ensures better fit-resulting, a more natural, and harmonious restoration of the skull’s shape. However, in order to print implants, the materials must possess suitable properties such as optimal viscosity for precise layer deposition for FDM technology, sufficient mechanical strength to withstand physiological loads, and thermal stability to maintain integrity during the printing process. Additionally, the materials should exhibit low shrinkage during cooling to ensure dimensional accuracy and customizability to tailor the implant to the patient’s specific anatomical needs.

Various treatment strategies are employed depending on the size, location, and severity of the defect. Traditional methods, such as autologous bone grafting, remain a standard approach, offering the advantage of biocompatibility and the potential for bone regeneration. However, limitations in graft availability, resorption rates, and complications like donor site morbidity often lead to the exploration of alternative solutions.

Apart from others, synthetic materials such as titanium, hydroxyapatite, and polymethyl methacrylate (PMMA) offer benefits like reduced surgical time, elimination of donor site morbidity, and more predictable outcomes for cranioplasty. Additionally, advances in 3D printing and personalized implants have revolutionized cranial defect repair, allowing for the production of custom-made prostheses tailored to individual patients, and enhancing the precision and esthetic outcomes of the procedure.

Furthermore, the field has witnessed a rise in tissue engineering and regenerative medicine approaches, including the use of stem cells and biocompatible scaffolds. These techniques aim to promote bone regeneration and repair by encouraging cellular growth and the formation of new bone tissue at the site of the defect. Studies investigating the use of growth factors and gene therapy are also underway, offering promising avenues to enhance the healing process and improve long-term outcomes.

While these novel treatments show great potential, challenges remain in terms of long-term efficacy, cost, and widespread clinical application. Research continues to focus on optimizing materials, improving surgical techniques, and ensuring the durability and safety of the treatments. In conclusion, the treatment of cranial bone defects is an evolving field, combining traditional surgical techniques with cutting-edge technologies, continually enhancing the quality of care for patients facing these challenging conditions.

## Figures and Tables

**Table 1:** Statement of selected examples of the use of implant materials in the treatment and reconstruction of cranial bone defects.

Research Type	Application	Material	Conclusions	Ref.
Original article: a case series.	Reconstruction of bone defects in the cranial vault.	Methyl methacrylate (PMMA).	Cranioplasty—intraoperative PMMA cement patterning.	[107]
Original article: a case report.	Reconstruction of a large skull bone defect after a complication of cranioplasty performed with titanium mesh.	Three-dimensional 3D polycaprolactone (PCL) implant.	The implant was biocompatible, well established at the implant site with successful osteointegration. After a one-year follow-up, the implant was shown to perform both protective and esthetic functions. No complications were observed.	[108]
Original article: a case series.	Cranioplasty of large skull bone defects after decompressive craniectomy.	The implant is based on polycaprolactone (PCL) and tricalcium phosphate (TCP) with the addition of autologous biological material in the form of bone marrow.	The (PCL-TCP) implant with the addition of stem cells and active growth factors had a positive effect on the patients’ health. The imaging studies performed after 8 and 20 months showed ossification in the defect area.	[109]
Original article: research.	The composite implants for the reconstruction of cranial and maxillofacial defects.	The scaffolds based on polycaprolactone (PCL) fiber mats and calcium phosphate cement (CPC) paste. The scaffolds manufactured using 3D printing (FDM—Fused Deposition Modelling technology).	High strength with simultaneous satisfactory flexibility of the (PCL-CPC) material was demonstrated. The proposed solution can be used for facial bone substitute implants.	[110]
Original article: research.	The design and numerical analysis of an implant for major cranial bone defects.	The cranial implant made of (PEEK) polyetheretherketone.	The numerical and clinical studies conducted for the proposed (PEEK) implant solution showed that the scaffold fulfills its function by providing durability, reconstruction and protection of the brain, and improved esthetics.	[73]
Original article: research.	The effect of the used manufacturing technology on craniofacial implants including degradation time.	The implants manufactured by 3D printing method (FDM—Fused Deposition Modelling technology), based on poly(L-co-D,L-lactide) (PLDLA) with the addition of hydroxyapatite nanoparticles and implants manufactured by injection molding based on (PLDLA).	The research showed that material degradation time is significantly affected by the choice of implant manufacturing technology. It was observed that 3D printed implants degraded faster than those that were manufactured by injection molding.	[111]
Original article: research.	The implants dedicated to mandibular bone defects.	Implants based on titanium and polyetheretherketone (PEEK).	The manufactured implants demonstrated biocompatibility and promoted osteointegration. The proposed treatment method enables bone regeneration, which, in retrospect, may have a positive impact on preventing mandibular bone atrophy caused by aging patients.	[112]
Original article: a case report.	The treatment of cranio-maxillofacial defects tailored to the individual patient’s functional and esthetic needs. High percentage of defects caused by excision of tumors.	The implants are based on polyetheretherketone (PEEK).	The high success rate of implanted treatment of bone defects with (PEEK) implants after tumor resection. This study showed no implant failures (ruptures, dislocations).	[113]
Original article: a case series and review of literature.	Reconstruction of Large Cranial Defects.	Titanium implants manufactured using the additive method.	The use of additively manufactured titanium implants to treat large cranial bone defects is considered a good solution. The titanium implants are appropriate for bone reconstruction.	[114]
Original article: research.	The reconstruction of a zygomatic bone defect with an incrementally manufactured implant.	The implant based on polyetheretherketone (PEEK), manufactured using method 3D printing (FFF—Fused Filament Fabrication technology).	The experimental studies have shown that the proposed implant solution, together with the appropriate choice of material, is capable of handling heavy loads as well.	[115]
Original article: research.	Development of a porous NiTi fixation plate for mandibular bone reconstruction.	Implant based on NiTi manufactured by selective laser melting.	The developed finite element model for the porous NiTi fixation plate showed, proper stress distribution and proper fit of the plate to the bone graft.	[78]

## Data Availability

No new data were created or analyzed in this study. Data sharing is not applicable to this article.
